# Succinate in innate immunity: linking metabolic reprogramming to immune modulation

**DOI:** 10.3389/fimmu.2025.1661948

**Published:** 2025-09-25

**Authors:** Reham Atallah, Juergen Gindlhuber, Akos Heinemann

**Affiliations:** ^1^ Otto Loewi Research Center for Vascular Biology, Immunology and Inflammation, Division of Pharmacology, Medical University of Graz, Graz, Austria; ^2^ Division of Cardiology, Medical University of Graz, Graz, Austria

**Keywords:** succinate, innate immune cells, succinate dehydrogenase (SDH), reactive oxygen species (ROS), hypoxia-inducible factor-1α (HIF-1α), succinylation, succinate receptor 1 (SUCNR1), inflammation

## Abstract

Succinate is an essential metabolite in the tricarboxylic acid (TCA) cycle. In mitochondria, succinate holds a unique position connecting the TCA cycle and the electron transport chain (ETC), thereby providing a shortcut path for adenosine triphosphate (ATP) production. Beyond this fundamental role in cellular metabolism, succinate is increasingly acknowledged as a key modulator of immune cell function. Production of reactive oxygen species (ROS), hypoxia-inducible factor-1α (HIF-1α) stabilization, protein succinylation and cell-cell communication mediated by succinate receptor 1 (SUCNR1) are traits induced by succinate. During inflammation, succinate plays key dual roles, culminating in either pro- or anti-inflammatory effects that are tissue- and context-dependent. In this review, we provide a succinct overview focusing on the regulatory role of succinate in innate immune cells, highlighting involved mechanisms and research gaps that represent promising targets for future study.

## Graphical Abstract

Key sources of elevated succinate, innate immune cell types and potentially associated diseases. Elevated succinate can arise from gut microbiota, metabolic rewiring, tissue damage and hypoxia. Depicted innate immune cell subsets potentially capable of sensing and responding to succinate include macrophages, dendritic cells, neutrophils, eosinophils, basophils, mast cells as well as natural killer and other innate lymphoid cells. Associated pathologies including gastrointestinal inflammation, allergic inflammation, ocular diseases, arthritis, cancer, obesity and obesity-induced diabetes, neuroinflammation, infection and sepsis are represented by disease icons, reflecting multifactorial processes involving multiple cell types. Arrows indicate the flow from succinate to innate immune cells and from these cells to the associated diseases. HIF-1α, Hypoxia-inducible factor-1α; O_2_, Molecular oxygen.

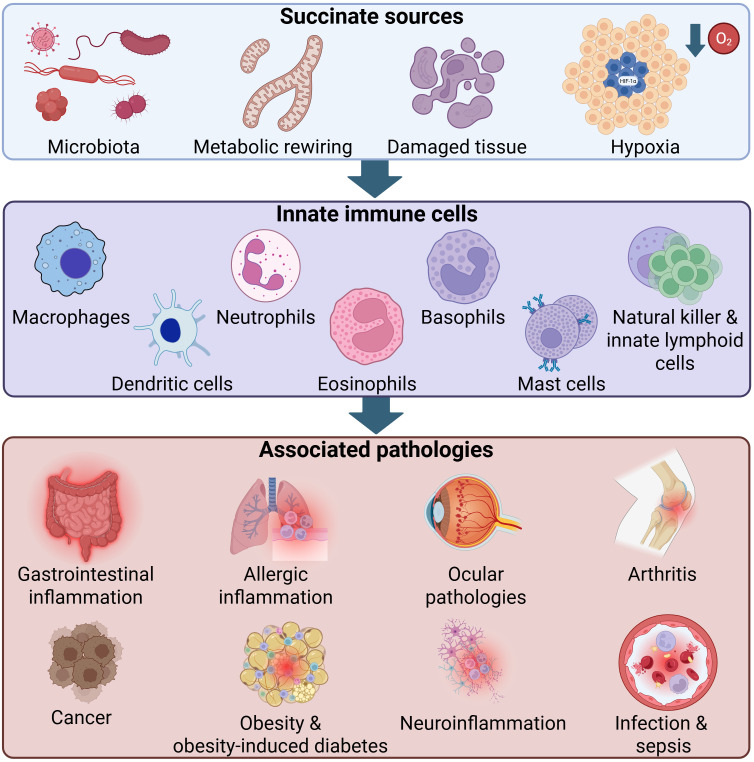

## 1 Introduction

In addition to physical and chemical barriers defending the host against foreign pathogens, the immune system encompasses two complementary lines of defense, i.e. innate and adaptive immunity (1). Unlike adaptive immunity, which is antigen-specific, slower to respond and provides immunologic memory, the innate immune system is non-specific in nature, fast and does not provide immunologic memory (2, 3). These two systems function in synchrony to ensure effective clearance of pathogens and minimize possible damage to host tissues (4). The adaptive immune system relies mainly on B and T cells (5), whereas phagocytes (macrophages and neutrophils), dendritic cells (DCs), eosinophils, basophils, mast cells (MCs), natural killer (NK) and other innate lymphoid cells (ILCs) orchestrate innate immunity (2, 6).

Recent advances in the field of immunometabolism unraveled a central role of immune cell metabolism in shaping the immune response (7). Indeed, innate immune cells undergo extensive metabolic reprogramming, upon exposure to external stimuli, which drives their activation state and phenotype (8). For instance, upregulation of glycolysis and the pentose phosphate pathway occurs concomitant to a reduction in oxidative phosphorylation in stimulated macrophages and DCs (9, 10). This metabolic switch facilitates rapid adenosine triphosphate (ATP) generation ensuring cell survival and provides biosynthetic precursors required for cytokine production (11–13). In line with global metabolic changes, individual metabolites such as succinate possess signaling ability and are able to modulate immune cell function (14).

In the tricarboxylic acid (TCA) cycle, succinate is produced from succinyl-coenzyme A (CoA) through the enzyme succinyl-CoA synthetase. Subsequently, succinate acts as a substrate for the enzyme succinate dehydrogenase (SDH, also known as complex II), producing fumarate and contributing to ATP production (15). Intracellular succinate accumulation has been reported in immune cells such as bone marrow-derived macrophages (BMDMs) stimulated with the bacterial membrane component lipopolysaccharide (LPS). This increase was attributed to increased glutamine-dependent anaplerosis and γ-aminobutyric acid (GABA) shunt (9). In addition, reduced or reverse SDH activity could result in succinate accumulation (14, 16). Another source of succinate could be the glyoxylate shunt, in which isocitrate is converted to succinate via the enzyme isocitrate lyase. Activity of this enzyme is increased under hypoxic conditions (17, 18), and is supposed to produce succinate to sustain the mitochondrial membrane potential and cell viability (19). Uptake of extracellular succinate serves as another source of succinate elevation in the cells and has been reported to suppress degranulation and production of interferon (IFN)-γ in T cells (20). Taken together, there are numerous sources that might contribute to succinate elevation in the cells under certain conditions, including immune cell activation.

Via distinct mechanisms, increased intracellular succinate could alter cell function and phenotype. By inhibiting prolyl hydroxylase domain (PHD) enzymes, succinate stabilizes the transcription factor hypoxia-inducible factor-1 alpha (HIF-1α) increasing the production of interleukin (IL)-1β and driving a pro-inflammatory phenotype in macrophages (9). In addition, reactive oxygen species (ROS) production driven by succinate oxidation contributes to this phenotype (21). Succinate can also modify proteins by succinylation of lysine residues altering their structure and function (22). An example of which is histone succinylation, which regulates gene transcription by weakening the affinity between deoxyribonucleic acid (DNA) and histones facilitating the binding of transcription factors to DNA (23). Conversely, extracellular succinate acts as a signaling molecule by engaging succinate receptor 1 (SUCNR1) and guiding immune cell responses, which might be pro- or anti-inflammatory depending on the cell type and context (24, 25). These divergent mechanisms underscore the central regulatory role of succinate. Importantly, these pathways are not independent and might act in synchrony to modulate cellular responses. For instance, activation of SUCNR1 can engage a phosphoinositide 3-kinase (PI3K)-HIF-1α axis that promotes tumor-associated macrophage polarization and cancer metastasis (26). Similarly, activation of SUCNR1 in human umbilical vein endothelial cells results in HIF-1α activation and increased IL-1β production (27). Herein, we provide a concise review of succinate involvement in innate immune cell function, discussing contributing downstream mechanisms and highlighting potential targets for future research and therapeutic opportunities.

## 2 Succinate as a regulatory metabolite

Succinate is a circulating metabolite and is detected in the circulation in the low µM range under steady-state conditions (28, 29). These concentrations may rise drastically under stress conditions like exercise (30), and in pathological conditions including hypertension (31), ischemic heart disease (32), obesity (33) and cancer (26, 34). The origin of circulating succinate is not completely clear. However, release by damaged or injured tissues and production by specific gut microbiota are highly plausible sources (35).

**Figure 1:**
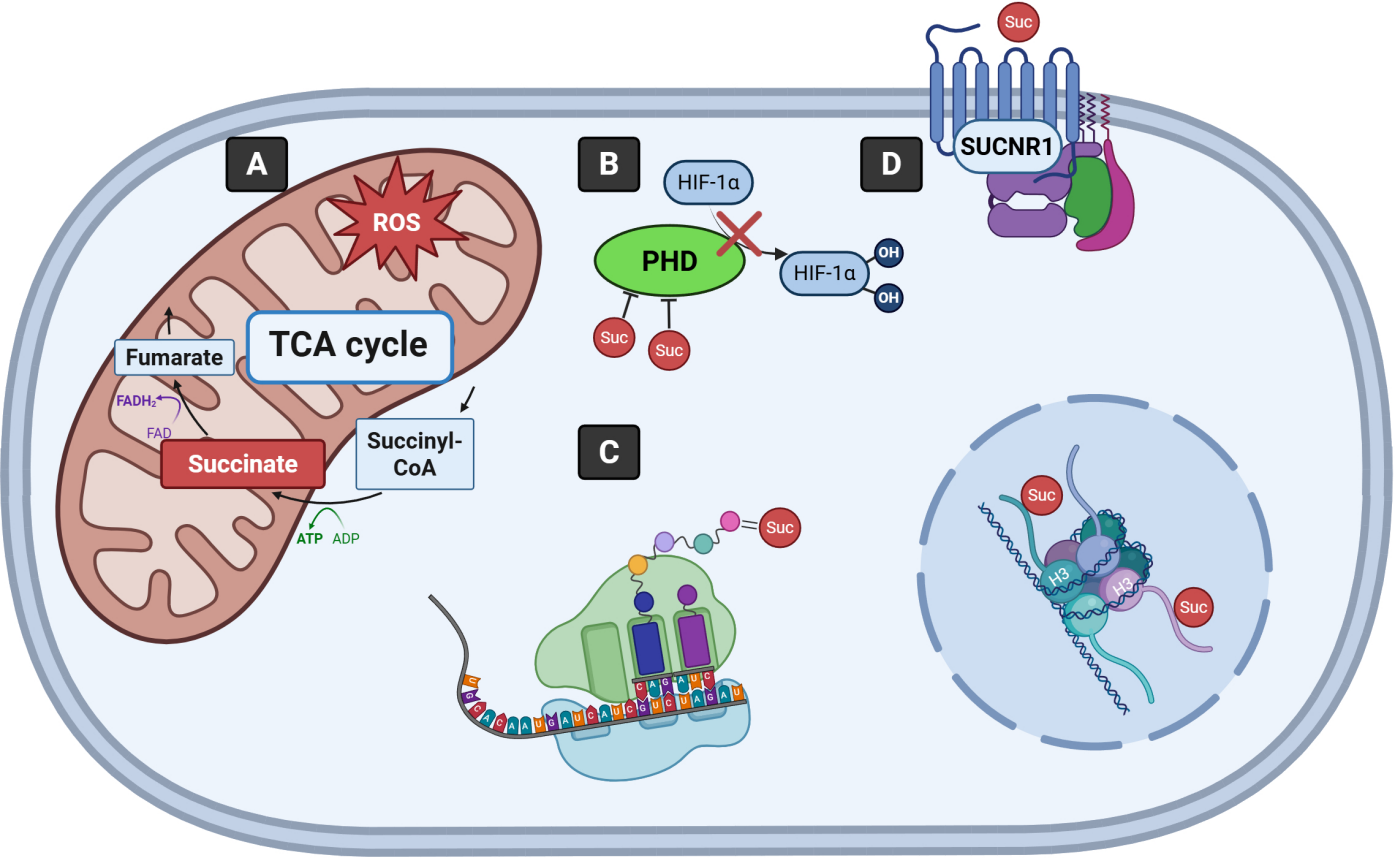
Signaling mechanisms driven by succinate. **(A)** Succinate is metabolized to fumarate as part of the TCA cycle in the mitochondria. Excessive succinate contributes to increased mitochondrial ROS production. **(B)** Succinate inhibits PHD enzymes, stabilizing HIF-1α by preventing its hydroxylation and degradation. **(C)** Succinylation of lysine residues of proteins, including histones, impacts their structure and function. **(D)** Succinate binding to SUCNR1 enables cells to sense and respond to extracellular succinate. TCA, Tricarboxylic acid; CoA, Coenzyme A; ADP, Adenosine diphosphate; ATP, Adenosine triphosphate; FAD, Flavin adenine dinucleotide; FADH_2_, Reduced flavin adenine dinucleotide; ROS, Reactive oxygen species; PHD, Prolyl hydroxylase domain; HIF-1α, Hypoxia-inducible factor-1α; H3, Histone H3; SUCNR1, Succinate receptor 1; Suc, Succinate.

At the cellular level, the permeability of membranes for succinate is limited by its charged nature necessitating cellular transporters for its transfer. Indeed, the dicarboxylate carrier, a member of the solute carrier transporter family 25 (SLC25), and the voltage-dependent anion channel facilitate succinate transfer from mitochondria to the cytosol (36, 37). Succinate can also be effluxed to the extracellular space via organic anion/dicarboxylate transporters and monocarboxylate transporter 1 (MCT1) (38, 39). In conditions of increased energy demand and excessive anaerobic energy metabolism, excessive lactate production results in cell acidification leading to succinate protonation and allowing it to cross cell membranes with MCT1 (30). Influx of succinate in the cells could be mediated via members of the SLC13 family as was described in neural stem cells (40). Additionally, a role of MCT1 in the uptake of succinate into CD4^+^ T cells was previously described (20). Likewise, MCT1 facilitates succinate import in murine brown adipocytes, an uptake that was pH-dependent (41). Based on its localization in the cell, succinate can modulate different cellular pathways altering cell phenotype and function, as will be discussed in this section. **Figure 1** summarizes these mechanisms.

### 2.1 Succinate metabolism and ROS production

In the mitochondria, succinate is metabolized to fumarate via SDH, a multi-subunit enzyme, which requires numerous proteins for its assembly (42). In eukaryotes, SDH is composed of four subunits, SDHA to SDHD, from which A and B represent the catalytic domain, while C and D are anchor proteins (42). During succinate oxidation by SDH, electrons are transferred to ubiquinone in the electron transport chain (ETC) and participate in ATP production (43). Over recent years, the roles of succinate and SDH in pathological conditions, particularly those involving immune cells functioning in hypoxic environments such as chronic inflammation, ischemia-reperfusion injury and cancer, have gained increasing attention, as comprehensively reviewed by Zhang and Lang (44).

Although complex I and complex III are considered the main sites for mitochondrial ROS production, increasing evidence indicates that SDH could be involved (45, 46). Indeed, mutations in subunit C of SDH are linked to oxidative stress, genomic instability and tumorigenesis in hamster fibroblasts and SDHC E69 mouse cell line (47, 48). These findings have been further validated in yeast studies, where gene deletion or mutation in SDH subunits results in increased ROS production (49–51). In addition, inhibition of SDH has been shown to reduce glucose-induced ROS production and insulin secretion in Langerhans islet cells from mice, confirming the regulatory role of SDH in ROS production and glycemic control (52). In a murine model of ischemia, succinate levels are elevated and are attributed to reversal of SDH, caused by fumarate overflow from purine nucleotide breakdown and the malate/aspartate shuttle. Upon reperfusion, metabolism of succinate via SDH is responsible for mitochondrial ROS production through reverse electron transport at complex I (53). In line with that, inhibition of SDH by intracoronary malonate during early reperfusion reduces reperfusion injury and infarct size in a pig model of transient coronary occlusion (54).

Notably, in human studies there is discrepancy in the results of studies investigating the role of SDH as a source of ROS and it is not clear whether this is due to biological or technical reasons. For instance, Guzy et al. have shown that pharmacological inhibition or ribonucleic acid (RNA) interference of SDHB, but not SDHA, in human Hep3B cells results in increased ROS production and HIF-α stabilization, a response that is ROS-dependent (46). Conversely, in SDHD-deficient human embryonic kidney (HEK) 293 cells, there is no indication of increased ROS production as compared to controls and HIF-1α stabilization in these cells is mostly mediated by succinate and is not ROS-dependent (55). Similar findings have been reported in SDHA-mutant fibroblasts (56). Taken together, ROS production represents an important signaling mechanism that could be driven by elevated succinate under certain conditions.

### 2.2 Stabilization of HIF-1α

Another mode by which succinate acts as a signaling molecule in the cytosol is via the inhibition of α-ketoglutarate-dependent dioxygenases (57). These enzymes include HIF-α-PHDs, which play a crucial role in regulating HIF stability (58). PHD enzymes use oxygen and α-ketoglutarate as substrate, and iron as well as ascorbate as co-factors to hydroxylate proline residues on HIF-α making it recognizable by von Hippel-Lindau (VHL) protein. Once bound by VHL protein, HIF-α is polyubiquitylated and degraded (58). PHDs produce succinate as a product and are, therefore, inhibited by the accumulated succinate (product inhibition) (59). In such cases, HIF-α is not hydroxylated and subsequently is not degraded, which can occur also under normoxia and is referred to as pseudohypoxia (29). HIF-α forms a heterodimer with HIF-β and the active complex in the nucleus drives the expression of genes involved in several processes such as angiogenesis, metabolism and cell survival (60). Another indirect mechanism by which succinate accumulation could stabilize HIF is via ROS production (46).

Among α-ketoglutarate-dependent dioxygenases are the ten eleven translocation (commonly known as TET) DNA demethylases, a group of enzymes that promotes DNA demethylation through oxidizing methylcytosines (61), and the Jumonji C domain-containing proteins, which have histone demethylase catalytic activity and thus are very important epigenetic modulators (62). Therefore, increased succinate levels could possibly influence the cellular epigenetic landscape, resulting in long-term consequences for gene expression (63).

### 2.3 Protein succinylation

Succinylation is another crucial signaling mechanism potentially driven by succinate (64). It denotes the incorporation of a succinyl group to lysine residues of proteins, thereby altering protein function (65, 66). In comparison to other post-translational modifications (PTMs) like methylation and acetylation, succinylation probably has a bigger impact on protein properties, given the larger size of succinate and the significant change in the charge of lysine by succinate from +1 to –1 (67). This process could occur both non-enzymatically and enzymatically (67), and takes place inside and outside the mitochondria (68). In non-enzymatic succinylation, succinyl-CoA acts as the succinyl donor (69), and succinate could serve as a source for this metabolite as has been shown in *Escherichia-coli* (70). Supporting this finding, a recent study has illustrated that succinate derived from microbiota increases succinylation of PurR, a transcription factor that negatively regulates purine biosynthesis genes, to enhance *Citrobacter rodentium* virulence in a mouse model of enterohaemorrhagic *Escherichia coli* (71). In enzymatic succinylation, lysine succinyl transferases regulate protein succinylation in the cells (72), as has been shown for carnitine palmitoyl transferase 1A (CPT1A), an important enzyme in fatty acid oxidation (73). The succinylase activity of CPT1A promotes cell proliferation under glutamine depletion (73). Likewise, the enzyme lysine acetyltransferase 2A might function as a succinyl transferase to succinylate histone H3, enhancing tumor cell proliferation and tumor growth (74). In line with this, a recent study revealed that high succinylation scores in colorectal cancer correlate with mitochondrial oxidative phosphorylation and ETC, while low succinylation scores associate closely with immune cell differentiation. Spatial transcriptomic analysis further demonstrated a negative correlation between succinylation scores and immune cell activity in tumor-adjacent regions, highlighting the potential role of succinylation in shaping the tumor-immune microenvironment and influencing immune surveillance and tumor progression (75).

In contrast, desuccinylation is a process that regulates the level of protein succinylation within cells. This process is primarily catalyzed by enzymes, among which members of the sirtuin (SIRT) family have been studied extensively (67). The SIRT family is a group of nicotinamide adenine dinucleotide (NAD^+^)-dependent lysine deacetylases that regulate important biological processes including metabolism (76). Mammals have seven sirtuins numbered from 1 to 7, with SIRT5 and SIRT7 having desuccinylase activity (77, 78). Numerous studies have addressed the impact of SIRT5 and SIRT7-mediated protein desuccinylation in physiological and pathological contexts and can be reviewed elsewhere (67, 79). As an example, SIRT5 suppresses SDH activity resulting in diminished cellular respiration and knockdown of SIRT5 increases SDH activity and cellular respiration in the presence of succinate (80). The interaction of SIRT5 with SDHA has been confirmed in another study to result in its desuccinylation, while knockdown of SIRT5 causes hypersuccinylation and reactivation of SDHA (81). SIRT7, on the other hand, is a histone desuccinylase that links chromatin condensation and genome stability, while SIRT7-mediated desuccinylation of histones enhances chromatin condensation and DNA repair (78). Collectively, there is a fine balance between succinylation and desuccinylation in cells and its maintenance is crucial for the regulation of cellular responses.

### 2.4 Signaling of SUCNR1

The identification of SUCNR1, previously known as G protein-coupled receptor (GPCR) 91 or GPR91, as a specific receptor for succinate in a landmark study by He et al. opened the door for extensive research on the role of this receptor in different cells and tissues in physiological and pathological contexts (82). It is postulated that SUCNR1 acts as a sensor to metabolic alterations caused by tissue stress and subsequently drives the tissue to respond. Therefore, dysregulated or excessive activation of this receptor might underlie pathological conditions. SUCNR1 is expressed in many organs including the kidneys, the spleen, the liver, the heart and the small intestine (82, 83). At the cellular level, SUCNR1 expression was evident in structural cells like endothelial cells (84), fibroblasts (85), cardiomyocytes (86) and adipocytes (87) as well as immune cells including macrophages (24, 25, 88) and DCs (89, 90). The activation of this receptor induces varying responses and is implicated in ischemia-reperfusion injury (91), hypertension (82, 92), immune response and inflammation (93–95), platelet aggregation (96), angiogenesis (29, 84, 97) and glucose homeostasis (98). As a GPCR, activation of SUCNR1 by succinate triggers downstream signaling pathways which are also cell type specific. For example, in HEK293 cells, succinate-mediated activation of SUCNR1 induces intracellular calcium release, accumulation of inositol triphosphate, activation of extracellular-signal-regulated kinases 1/2 (ERK1/2) and a decrease of cyclic adenosine monophosphate (cAMP) concentration, which indicates that SUCNR1 couples to both a pertussis-toxin-sensitive Gi/Go pathway and a pertussis-toxin-insensitive Gq pathway (82). In contrast, succinate increases, rather than decreases cAMP, in cardiomyocytes resulting in protein kinase A activation, suggesting SUCNR1 coupling to Gs (32). These distinct signaling pathways triggered by SUCNR1 activation emphasize that succinate actions are diverse and complex and require in-depth investigation.

## 3 Succinate in innate immune cells

The innate immune system is comprised of four defense barriers including anatomic barriers (skin and mucous membranes), physiologic barriers (temperature, pH and chemical mediators), endocytic and phagocytic barriers, and inflammatory barriers (1). Innate immunity relies on a group of specialized immune cells such as phagocytes (macrophages and neutrophils), DCs, eosinophils, basophils, MCs, as well as NK and other ILCs (2). Unlike B and T cells, innate immune cells lack antigen-recognition receptors (1). Nonetheless, they recognize and bind specific microbial molecular structures known as pathogen-associated molecular patterns (PAMPs), in addition to tissue-derived damage-associated molecular patterns (DAMPs) through the germline-encoded pattern recognition receptors (PRRs) (99). Examples of PAMPs are the bacterial product LPS and viral double-stranded RNA (100, 101), while DAMPs include biglycan, histones and heat-shock proteins, among others (102). PRRs are expressed on the cell surface as well as intracellularly and include toll-like receptors (TLRs), C-type lectin-like receptors (CLRs) and Nod-like receptors (NLRs) (103).

**Figure 2:**
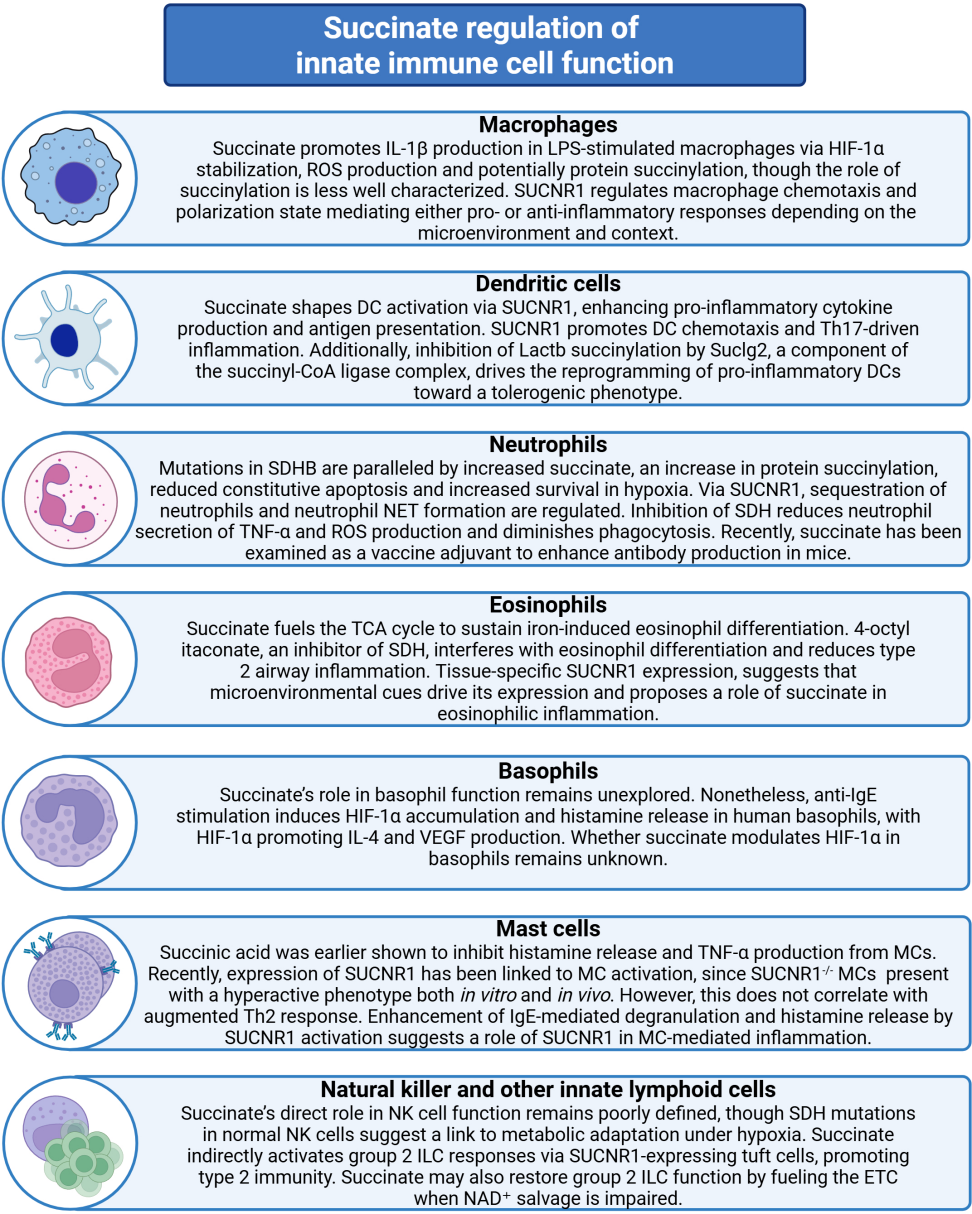
Succinate regulation of innate immune cell functions. Illustration of innate immune cells accompanied by annotations summarizing key research findings on how altered succinate levels influence their functions. Relevant involved mechanisms are also indicated. SDHB, Succinate dehydrogenase subunit B; SDH, Succinate dehydrogenase; SUCNR1, Succinate receptor 1; NET, Neutrophil extracellular trap; TNF, Tumor necrosis factor; ROS, Reactive oxygen species; IL, Interleukin; LPS, Lipopolysaccharide; HIF-1α, Hypoxia-inducible factor-1α; DC, Dendritic cell; Th, T helper; Lactb, Lactamase beta; Suclg2, Succinate-coenzyme A ligase subunit beta; MC, Mast cell; IgE, Immunoglobulin E; TCA, Tricarboxylic acid; VEGF, Vascular endothelial growth factor; NK, Natural killer; ILC, Innate lymphoid cell; ETC, Electron transport chain; NAD^+^, Nicotinamide adenine dinucleotide.

At the site of infection or injury, innate immune cells produce cytokines and chemokines, which initiate both local and systemic responses (1). Indeed, the innate immune system drives a local inflammatory response, while simultaneously activating the adaptive immune system for subsequent response (2). Dysregulated innate immune response has been implicated in the development of autoimmune and inflammatory diseases such as lupus erythematosus and Sjögren syndrome (104). With the rise of the field of immunometabolism, it has become clear that metabolism of innate immune cells is central to driving their activation, differentiation and fate (7). In the following section, we will discuss the role of succinate in modulating the function of innate immune cells and underlying mechanisms will be addressed. A schematic overview is provided in **Figure 2**.

### 3.1 Macrophages

Macrophages represent a vital cellular component of the innate immune system. They serve three main functions, namely phagocytosis, antigen presentation and immune modulation (105). In addition, macrophages play an important role in iron homeostasis, tissue injury repair and other metabolic functions (106–108). Bone marrow-derived monocytes are the precursors of macrophages. They circulate in the blood for 1 to 2 days, then they are either recruited to tissues for differentiation or they die (109). Nonetheless, many tissue-resident macrophages like Kupffer cells in the liver and microglia in the brain develop from cells of embryonic origin and are seeded in different tissues before birth (110, 111). It is reported that some tissue-resident macrophages are capable of self-renewal in the tissue (112).

The functional destiny of macrophages is linked to their polarization state and is determined by environmental cues (109). Classically, macrophages have been classified into M1 and M2 macrophages. However, current understanding suggests that this classification is over-simplified and does not reflect the complex macrophage dynamics and plasticity *in vivo* (113). M1 macrophages produce high levels of toxic effector molecules such as ROS and nitric oxide (NO) and release pro-inflammatory cytokines including IL-1β, tumor necrosis factor (TNF) and IL-6. They also promote T helper (Th) 1 responses, and have microbicidal and tumoricidal activity (114). M2 macrophages, in contrast, release extracellular matrix components, angiogenic and chemotactic factors, as well as IL-10 (115). Therefore, they participate in polarized Th2 responses, clearance of parasites, tissue remodeling, angiogenesis, immunoregulation, allergy and tumor promotion (115). *In vitro*, macrophages can be polarized towards an M1 phenotype using microbial products like LPS and cytokines like TNF-α or IFN-γ, either alone or in combination (113), while M2 polarization can be induced using Th2 cytokines like IL-4 or IL-13 (116).

Among all cells of the innate immune system, macrophages are perhaps the most studied ones in terms of metabolic control of their phenotype and function (117–119). When stimulated by LPS, macrophages switch their metabolism from oxidative phosphorylation to glycolysis, which is a faster but less energy efficient means for ATP production (9). Alongside this metabolic switch, macrophages accumulate succinate and increase their production of pro-inflammatory cytokines such as IL-1β (9). Interestingly, accumulated succinate in BMDMs is sensed by intracellular pathogens such as *Salmonella Typhimurium* to drive their virulence and survival by facilitating antimicrobial resistance and the promotion of type-III secretion (120).

Different mechanisms seem to govern the pro-inflammatory macrophage phenotype driven by succinate. One such mechanism is succinate oxidation via SDH and increased ROS production (21). In BMDMs, inhibition of SDH using a cell permeable dimethylmalonate reduces LPS-mediated IL-1β production and increases IL-1 receptor antagonist and IL-10 (21). In this context, inhibition of SDH by itaconate controls succinate levels in LPS-activated macrophages and drives an anti-inflammatory phenotype measured as reduced IL-12, IL-6 production and inducible nitric oxide synthase (iNOS) expression (121). Another important mechanism by which succinate regulates IL-1β production in macrophages is via HIF-1α stabilization (9). Like succinate, dimethyloxallyl glycine, an inhibitor of PHD, boosts LPS-induced Il-1β gene expression, while α-ketoglutarate supplementation abolishes it (9).

Besides ROS production and HIF-1α stabilization, succinylation is another potential mechanism driving succinate function in macrophages. Notably, succinylation of many proteins in response to LPS stimulation has been reported in macrophages in the study of Tannahill and colleagues (9). However, the implications of this PTM in relation to the observed macrophage phenotype was not addressed. In addition, SIRT5-mediated desuccinylation of pyruvate kinase M2, a critical enzyme in glycolysis, reduces IL-1β production in LPS-activated macrophages (122). Furthermore, inhibition of SIRT2 by low concentrations of NAD^+^ results in the accumulation of acetylated α-tubulin, which in turn mediates the assembly of NOD-like receptor pyrin domain containing 3 (NLRP3) inflammasome and drives IL-1β production in BMDMs (123). Deletion of SIRT1 in macrophages results in hyperacetylation of nuclear factor kappa-light-chain-enhancer of activated B cells (NF-κB), thereby increasing the activation of NF-κB-dependent proinflammatory targets such as TNF-α, IL-1β, manganese superoxide dismutase and cellular inhibitor of apoptosis 2 in response to TNF-α stimulation (124).

The engagement of SUCNR1 in succinate-mediated responses in macrophages is also evident, however, mediating controversial responses. Indeed, SUCNR1-mediated macrophage chemotaxis has been implicated in obesity-induced diabetes (24). Release of succinate from adipose tissue in response to hypoxia and hyperglycemia drives macrophage chemotaxis to adipose tissue inducing inflammation and glucose intolerance (24). In SUCNR1^-/-^ mice, significantly less macrophages infiltrate the adipose tissue as compared to wild type and SUCNR1^-/-^ mice remain glucose tolerant (24). Likewise, cancer cells produce succinate which drives macrophage migration via SUCNR1, a response that is abrogated by an anti-SUCNR1 antibody (26). Accumulation of microglia in the retina of SUCNR^-/-^ mice relative to control mice suggests a role of SUCNR1 in the pathogenesis of age-related macular degeneration (125). While the motility of SUCNR1^-/-^ microglia is compromised, a global deficiency of SUCNR1 was required to observe the phenotype in mice proposing that the role of SUCNR1 in microglia is probably dispensable (125).

In addition, the role of SUCNR1 in driving macrophage polarization has been confirmed by recent evidence but has also yielded contradicting results. While expression of SUCNR1 by M2 macrophages is, in fact, significantly higher than by M1 macrophages, subsequent activation of M2 macrophages with succinate or compound 131 (SUCNR1 agonist) skews them to a pro-inflammatory phenotype with increased TNF-α and reduced IL-10 expression (126). Furthermore, stimulation of murine BMDMs with LPS results in increased IL-1β gene expression, a response that is hampered in SUCNR1-deficient cells (127). This finding indicates a pro-inflammatory role of SUCNR1 in macrophages. Notably, stimulation of BMDMs with IL-1β increases SUCNR1 expression proposing a positive-feedback loop that drives chronic inflammation. In this setting, SUCNR1^-/-^ mice show reduced macrophage activation and IL-1β production in a model of antigen-induced arthritis (127).

This pro-inflammatory view of SUCNR1 in macrophages has been challenged by a recent study showing that myeloid-specific lack of SUCNR1 results in increased expression of pro-inflammatory genes (*Il1b, Il12b, Tnf and Nos2*), particularly in white adipose tissue, together with increased number of CD11b^+^CD11c^+^CD206^-^ pro-inflammatory macrophages (25). Furthermore, SUCNR^-/-^ BMDMs increase their production of IL-6, TNF-α and NO in response to stimulation with LPS or LPS+INF-γ stimulation (128). In cancer, succinate-SUCNR1 signaling governs anti-inflammatory tumor-associated macrophage polarization as indicated by increased expression of arginase 1 (*Arg1*)*, Fizz1* (also known as *Retnla*), macrophage galactose-type lectin 1 (*Mgl1*) and macrophage galactose N-acetyl-galactosamine specific lectin 2 (*Mgl2*) upon treatment of peritoneal macrophages *in vitro* with succinate (26). In a syngeneic murine tumor model, succinate-treated mice have a significantly increased number of VCAM1^+^CD11c^+^CD11b^low-^ tumor-associated macrophages than control mice (26). Further data show a role of SUCNR1 in ameliorating chronic neuroinflammation *in vivo* (40). In this study, succinate released by pro-inflammatory macrophages activates SUCNR1 in neural stem cells driving them to increase their production of prostaglandin E2 and scavenging extracellular succinate, which consequently contributes to the resolving of inflammation (40). Together, succinate plays a crucial role in driving macrophage phenotype and function, culminating in either pro- or anti-inflammatory activity. Several factors, including tissue context and the exact cellular mechanisms driven by succinate, appear to be major determinants in either response.

### 3.2 Dendritic cells

Based on their phenotype and functionality, DCs can be categorized into major subsets such as conventional DCs, monocyte-derived DCs, plasmacytoid DCs and Langerhans cells (129). These subpopulations have different migratory abilities, follow different migratory paths and drive distinct immunological and inflammatory responses (129). As the master antigen presenting cells, DCs detect pathogens through their PRRs (130). Once stimulated, DCs mature and migrate to secondary lymphoid organs where they interact with T cells driving their activation, expansion and differentiation into effector T cells (131). DC maturation implies the redistribution of major histocompatibility complex (MHC) molecules to cell surface, a reduction in endocytic capacity and a pronounced increase in expression of co-stimulatory molecules such as CD80 and CD86 (132, 133). Notably, DCs also undergo profound changes in their morphology and re-organize their cytoskeleton (134). By presenting complete unprocessed antigens on their surface, DCs can stimulate B cells to initiate an antigen-specific antibody response (135). Another essential function of DCs is immune tolerance. Indeed, immature DCs constantly present self-antigens and non-pathogenic antigens to T cells to sustain immune tolerance via different mechanisms including the differentiation of regulatory T cells (known as Tregs), T cell deletion and induction of T cell anergy (136).

Resting DCs are able to use both glycolysis and mitochondrial respiration to meet their metabolic demands (11). Like macrophages, DCs undergo striking metabolic changes upon stimulation with TLR agonists switching their metabolism to favor glycolysis (10). This metabolic switch is essential for their survival as oxidative phosphorylation and ATP production are reduced by increased NO production (11). In addition, this glycolytic flux is essential for the *de novo* synthesis of fatty acids required for the expansion of cellular organelles responsible for the production and secretion of proteins that are crucial for DC activation (137). Recently, succinate-CoA ligase subunit beta (Suclg2) has been identified as a key metabolic enzyme in the reprogramming of pro-inflammatory mature DCs into a tolerogenic phenotype (138). Suclg2 inhibits the succinylation of the mitochondrial protein lactamase beta (Lactb), which subsequently results in reduced NF-κB signaling activation (138).

In comparison to other immune cells, immature DCs express relatively high SUCNR1 transcripts (89). Activation of SUCNR1 drives DC chemotaxis and in synergy with TLR-3 and TLR-7, but not TLR-2 or TLR-4, SUCNR1 potentiates the expression of proinflammatory mediators like TNF-α and IL-1β (89). This response culminates in enhanced antigen presentation and activation of CD4^+^ T cells (89). *In vivo*, SUCNR1 mediates DCs chemotaxis into draining lymph nodes, subsequently driving the expansion of Th17 cells, which contribute to autoimmunity. Therefore, SUCNR1^-^/^-^ mice show reduced inflammation in an experimental arthritis model (90). These findings highlight SUCNR1 as an important target regulating the crosstalk between innate and adaptive immune cells during immune-mediated inflammation.

Of interest, Inamdar and colleagues have used succinate-based polymers to induce a pro-inflammatory phenotype in DCs by modulating their metabolism (139). In mice, administration of succinate polymer drives a significant pro-inflammatory anti-melanoma response, thereby offering an approach for developing antitumor metabolite-based therapies (139). Overall, the crucial role of succinate in dictating DC phenotype and function might represent a new frontier to modulate undesired inflammatory responses.

### 3.3 Neutrophils

Neutrophils, the most abundant circulating leukocytes in humans, are the first responder immune cells in case of infection or injury (140). They are produced from myeloid precursors in the bone marrow and are generally viewed as short-lived cells that circulate in the blood for few hours (141). Nonetheless, it has been shown that neutrophil lifespan in the blood could be much longer (142). Neutrophils migrate to sites of inflammation in response to chemotactic signals, where they phagocytose microorganisms and kill them using different mechanisms such as NADPH-dependent ROS production and release of antibacterial proteins such as cathepsins and defensins from their granules (142). To combat extracellular pathogens, they also release neutrophil extracellular traps (NETs), which are composed of DNA, histones, proteins like lactoferrin and enzymes like myeloperoxidase and elastase (143). Besides their indispensable role in acute inflammation, a role of neutrophils in chronic inflammation and adaptive immunity is increasingly appreciated (144, 145). These expanding functions reflect the complexity of neutrophils and the presence of heterogeneous neutrophil subpopulations adds another layer of complexity to these cells (146).

Emerging evidence gradually unfolds the metabolic flexibility of neutrophils, with the ability to rewire their metabolism upon stimulation to perform distinct functions. As an example, neutrophils switch to pentose phosphate pathway during oxidative burst, which becomes the main pathway for glucose metabolism (147). This flexibility is highly relevant since it was traditionally believed that neutrophils rely exclusively on glycolysis (148), with the activity of mitochondria being very limited except to drive apoptosis (149, 150).

Alongside this development in our understanding of the metabolic adaptations of neutrophils, the regulatory role of succinate in neutrophil function is increasingly appreciated. Indeed, peripheral blood neutrophils isolated from patients with heterozygous germline mutations in SDHB accumulate more succinate relative to controls (151). This is paralleled by an increase in protein succinylation, reduced constitutive apoptosis and increased survival in hypoxia, a phenotype that is not dependent on HIF-1α but is mostly linked to impairment of SDH and reduced oxidative stress (151). Similarly, circulating neutrophils from cystic fibrosis patients increase their glycolysis (Warburg effect) as indicated by increased succinate levels, subsequent HIF-1α stabilization and increased pro-IL-1β production. Of note, mature IL-1β is only increased in neutrophils from bronchoalveolar lavage fluid of patients and is driven by the NLRP3 inflammasome via caspase-1 (152). Succinate is also significantly increased in plasma of acute respiratory distress patients and contributes to the sequestration of neutrophils to the lung via SUCNR1 (153). In contrast, inhibition of neutrophil infiltration by succinic acid is associated with amelioration of concanavalin A-induced acute liver injury in mice (154). A role of succinate signaling via SUCNR1 in experimental autoimmune uveitis is linked to increased neutrophil NET formation by succinic acid, a response that can be reversed by SUCNR1 antagonism (155). NETs can enhance a Th1/Th17 cell immune response characterized by elevated IFN-γ and IL-17A production (155). Furthermore, inhibition of SDH by dimethylmalonate inhibits *in vivo* neutrophil secretion of TNF-α and ROS production as well as diminished phagocytosis in a thioglycolate broth-induced neutrophil peritonitis model (156). Recently, an interesting study examined succinate as a vaccine adjuvant to enhance antibody production in mice (157). By increasing neutrophil recruitment to the immunization site and increased expression of neutrophil-derived B cell-activating factor, succinate offers a novel mechanism in immunological enhancement (157). Further studies following a similar approach, exploring succinate and its derivatives, may open new avenues to modulate neutrophil-mediated immunity.

### 3.4 Eosinophils

Eosinophils are produced in the bone marrow from pluripotent progenitors and migrate to the circulation as mature cells (158). They spend relatively short time in the circulation, around 18 h, before they migrate to peripheral tissues under steady-state conditions or to inflammatory sites guided by IL-5 and eotaxin-1 (CCL11), amongst others (159). Upon stimulation, eosinophils release granule proteins, including major basic proteins, eosinophil cationic protein, eosinophil peroxidase and eosinophil-derived neurotoxin (160). In addition, eosinophils have the ability to store and release both Th1 and Th2 regulatory cytokines, which are differentially released in response to distinct stimuli (161). The role of eosinophils in type 2 immune responses marked them as crucial players in atopic diseases like asthma and allergy and in helminthic infections (162, 163). Eosinophils also play a role in antiviral immunity as they express TLRs associated with antiviral response both on their surface and intracellularly (164). Stimulation of these receptors drives eosinophil degranulation and, similar to neutrophils, release of DNA traps which contribute to viral clearance (164). Over the last years, eosinophils have been increasingly appreciated for a rather different role in maintaining tissue homeostasis mainly in the gastrointestinal tract, lungs, adipose tissue, thymus, uterus and mammary glands (165). Depending on the tissue, eosinophils pursue a crucial role in immunoregulation, glucose homeostasis, protection against obesity, preparation of the uterus for pregnancy and mammary gland development (165). The exact role of eosinophils in cancer remains unclear with conflicting results suggesting both tumorigenic and anti-tumorigenic roles (166–168). These distinct and possibly opposing effector functions are mediated by different eosinophil phenotypes including progenitor, circulatory, and tissue resident eosinophils (169).

While eosinophils and neutrophils show comparable glycolytic capacity, eosinophil mitochondrial respiration is significantly higher as indicated by increased oxygen consumption rate, maximal respiratory capacity and spare respiratory capacity (170). In response to stimulation with phorbol-myristate-acetate, a more sustained increase in oxygen consumption in eosinophils occurs relative to neutrophils (170). It is therefore plausible that eosinophils exhibit more metabolic flexibility as compared to neutrophils enabling them to adapt to diverse roles in different environments.

The role of succinate in eosinophil differentiation is only recently starting to unravel. Indeed, succinate levels increase in activated eosinophils, a metabolic shift aligning with elevated iron levels. Here, succinate fuels the TCA cycle to sustain iron-induced eosinophil differentiation (171). Another study shows that 4-octyl itaconate interferes with eosinophil differentiation and reduces type 2 airway inflammation (172). While inhibition of SDH by itaconate is established (121, 173), it has not been addressed in this study and thus a direct role of succinate in the observed responses remains obscure. In addition, expression of SUCNR1 in oesophageal-specific eosinophils but not peripheral blood eosinophils suggests a role of the local microenvironment in driving its expression. This assumption is corroborated by the substantial increase in SUCNR1 expression in peripheral blood eosinophils upon co-culture with oesophageal epithelial cells (174). Notably, the gene expression of succinate-metabolizing enzymes is dysregulated in the esophagus of patients with eosinophilic esophagitis relative to controls proposing a functional role of succinate in allergic eosinophilic responses (174). These few studies shed light on succinate as a valid target to explore in the context of eosinophilic inflammation.

### 3.5 Basophils

As the rarest circulating leukocyte population, basophils are produced in the bone marrow from progenitor cells (175). They have a short life span of 1 to 2 days (176). Basophils contain cytoplasmic granules and are able to release both pre-stored and newly synthesized pro-inflammatory molecules such as histamine, leukotrienes and cytokines such as IL-3, IL-4 and IL-13, which are critical in the development of allergy and hypersensitivity (177). In addition, basophils express high affinity immunoglobulin (Ig) E receptors (FcϵRI), whose aggregation occurs upon crosslinking of adjacent IgE molecules by bound allergen (178). This triggers basophils to degranulate and subsequently drives increased vascular permeability and tissue swelling in IgE-dependent anaphylactic response (179). Basophils also express TLRs, among which TLR4 is linked to exacerbation of allergic inflammation post infection (180, 181). Notably, basophils play a major role in immune modulation since infiltration of inflamed tissues at sites of allergic inflammation by basophils is usually associated with Th2 response (177). Indeed, increasing evidence suggests that basophils have the ability to function as antigen presenting cells and are able to induce a Th2 response to allergens and helminths (182–184). Furthermore, activation of basophils by autoreactive IgE skewing the immune system towards Th2 environment could influence the production of autoantibodies and thus contribute to the development of autoimmune diseases such as systemic lupus erythematosus (185, 186). Therefore, there is increasing appreciation of the role of basophils not only as effector cells driving inflammation but also as immunomodulatory cells bridging innate and adaptive immunity.

Currently, specific studies delineating the immune-metabolic adaptations or the modulatory role of succinate in basophil function are lacking. Nonetheless, it has been shown that accumulation of HIF-1α and histamine release occur in response to anti-IgE stimulation of primary human basophils (187). Upregulation of HIF-1α contributes to IgE-induced production of IL-4 and VEGF (187). Among these responses, IL-4 production is differentially regulated upon pre-stimulation with TLR-2 or TLR-4 ligands (188). Whether succinate is involved in HIF-1α accumulation in this case is not known.

### 3.6 Mast cells

In contrast to basophils, MCs are tissue-based cells located mainly at mucosal and connective tissues (178). They develop from haematopoietic progenitor cells released from the bone marrow and only differentiate in the tissue (189). MCs have a longer life span of weeks to months (177). Similar to basophils, MCs express FcϵRI and hence are important in IgE-mediated allergic responses (190). MCs also express other receptors including TLRs (191). They have cytoplasmic granules that contain histamine, proteases, growth factors and cytokines including TNF-α (192). It is noteworthy that MCs are probably the only cells storing pre-formed TNF (177). The role of this cytokine in the modulation of neutrophil influx during infection highlights the importance of MCs in the regulation of innate immunity against infection (193). Further, MCs can regulate adaptive immunity by secreted products like histamine, which alter the cytokines produced by DCs and subsequently driving a Th2 phenotype (194, 195). Another study showed that MCs prime DCs to promote a Th1 and Th17 phenotype (196). In addition to these regulatory functions, MCs are increasingly acknowledged for their role in extracellular matrix remodeling and angiogenesis (197–199). MCs are heterogeneous and are categorized into 2 subgroups based on the expression of key granule-associated proteases into tryptase- or tryptase and MC-specific chymase-expressing cells. These subtypes show distinct phenotypic characteristics and anatomic locations (192).

The data available on MC metabolic rewiring during development and activation, despite being relatively more abundant than that for eosinophils and basophils, remain limited. There is evidence that MCs undergo distinct metabolic shifts during IgE- and non-IgE-mediated activation (200). The shift towards glycolysis is indeed more prominent in non-IgE pathways (201), while mitochondria, via different mechanisms, modulate FcϵRI-mediated MC activation (202, 203).

The role of succinic acid in MCs has been examined in an early study, which illustrated that succinic acid has an inhibitory effect on MCs (204). Indeed, succinic acid inhibits histamine release from MCs stimulated with compound 48/80 or dinitrophenyl IgE and inhibits dinitrophenyl IgE-induced TNF-α production. Interestingly, the concentration of succinic acid required to inhibit TNF-α is lower than that required to inhibit degranulation suggesting different regulatory mechanisms. The stimulation with succinic acid results in an increase in cAMP levels which might be underlying the observed inhibition (204). Only recently, the expression of SUCNR1 was linked to MC activation, since MCs from SUCNR1^-/-^ mice present with a hyperactive phenotype both *in vitro* and *in vivo* (205). This hyperactivity does not correlate with augmented Th2 response measured as T cell infiltration and IL-4 and IL-13 production. While SUCNR1^-/-^ mice had increased allergic contact dermatitis reaction, this does not contribute to asthma or arthritis progression (205). In this study, the authors suggest that succinate signaling is essential for normal MC differentiation. However, this requires further investigation. The role of SUCNR1 in MC activation was further addressed in a study by Tang et al. who showed the activation of SUCNR1 in MCs from the umbilical cord or the MC line LAD-2 enhances IgE receptor-mediated degranulation and histamine release (206). This activation is mediated by SUCNR1/protein kinase C/ERK signaling pathway and potentiates antigen‐induced bronchoconstriction (206). These few studies establish a role of succinate and SUCNR1 in MC-mediated inflammation.

### 3.7 Natural killer and other innate lymphoid cells

NK cells belong to ILCs, which are a heterogeneous group of cells that derive from lymphoid lineage but lack genetically rearranged antigen receptors (207). ILCs are categorized into different subgroups based on the expression of key transcription factors and their cytokine production panel into NK cells, group 1 ILCs, group 2 ILCs, group 3 ILCs and lymphoid tissue-inducer cells (208).

NK cells play a crucial role in the control of viral infections and cancer (209). They represent around 5-15% of circulating blood cells and are also present in peripheral tissues like the liver, the placenta and the peritoneal cavity (210, 211). While resting NK cells circulate in the blood, they extravasate and infiltrate most tissues that are either infected with pathogens or have malignant cells (212, 213). To avoid attacking self-cells, NK cells express inhibitory receptors for self MHC-I molecules (214). Upon activation, NK cells utilize different mechanisms to pursue their effector functions including exocytosis of perforin/granzyme-containing granules, death-receptor-induced apoptosis and IFN-γ production (215, 216). The role of NK cells in regulating the function of other immune cells is evident in their cross-talk with DCs modulating T cell function (217). The detailed functions of other ILC subsets in immunity and in tissue homeostasis can be reviewed elsewhere (218).

Recently, the importance of metabolism in NK cell function started to unfold. Indeed, resting NK cells utilize glucose to maintain low levels of glycolysis and oxidative phosphorylation (219). Upon activation, NK cells undergo significant metabolic reprogramming as illustrated by increased glucose uptake and glycolysis, which is required for IFN-γ production and granzyme B expression (220). Furthermore, in cytokine-activated NK cells, the increased rate of oxidative phosphorylation is associated with increased mitochondrial mass (221). We refer the reader to other interesting reviews detailing the metabolic characteristics of NK cells and other ILC subsets in health and disease (222, 223).

To date, the number of studies that have addressed a link between succinate and ILC function is rather limited. An earlier study detected substantial levels of SDHB transcripts with a recurrent R46X mutation in normal mononuclear blood cells, with NK cells and monocytes being the main source of the mutant transcripts. In this study, the authors propose that this mutation, leading to downregulation of SDH function, might be a mechanism to facilitate early detection of, and pre-adaptation to, hypoxia (224). Yet, no other studies have investigated in detail how succinate elevation might drive a phenotypic change in NK cells and by which mechanism.

Indirect activation of group 2 ILCs in the intestine subsequent to succinate sensing by SUCNR1-expressing tuft cells has been demonstrated in a study by Nadjsombati et al. (225). In this context, succinate in the intestine, which can be produced by *Tritrichomonas protists* or bacterial microbiota drives a type 2 immune response via a circuit that includes tuft cells and group 2 ILCs (225–227). Nonetheless, succinate alone was unable to activate group 2 ILCs (225). In line with this, subsequent to mechanical injury and subcutaneous immunization, succinate release into the circulation from injured tissue drives intestinal inflammation characterized by tuft cell expansion and increased IL-25 culminating in increased group 2 ILCs and a propagated type 2 immune response (228). Recently, it has been shown that succinate, by feeding the ETC, is able to rescue group 2 ILC function upon genetic ablation or inhibition of nicotinamide phosphoribosyl transferase, the rate-limiting enzyme in the NAD^+^ salvage pathway (229).

## 4 Concluding remarks and future perspectives

While numerous studies have established succinate as a critical regulator of macrophage and DC function, its influence on other innate immune cell populations, particularly basophils, NK and other ILCs, remains largely unexplored. Elucidating succinate’s role in shaping the activity of these cells could uncover novel strategies for therapeutic targeting, especially in diseases characterized by excessive or dysregulated immune responses.

Beyond receptor signaling, mitochondrial SDH has emerged as a significant yet underestimated modulator of ROS production in diverse patho-(physiological) contexts. Manipulating SDH activity and developing targeted antioxidants to modulate cell phenotype and function present promising opportunities for conditions in which ROS plays a major pathogenic role, such as cancer and ischemia-reperfusion injury. Likewise, succinylation remains poorly understood and its potential interplay with other PTMs, such as acetylation, which might share regulatory pathways and functional consequences, warrants deeper investigation.

Despite growing interest in SUCNR1, the complexity of its signaling pathways and functional outcomes remains incompletely defined. SUCNR1 holds potential as an innovative drug target, and the development of selective small-molecule modulators will be key to fully characterize its contribution to health and disease.

Importantly, the potential synergistic or antagonistic interplay between the pathways regulated by succinate warrants careful study. Intracellular effects, such as PHD inhibition and HIF-1α stabilization, and extracellular SUCNR1-mediated signaling can converge to amplify inflammation or, under different conditions, counterbalance one another to restore homeostasis. The exact timing of pathway engagement, together with the prevailing metabolic and inflammatory milieu, is likely to critically influence the direction and magnitude of these effects. Understanding this context dependency will be essential to ensure that therapeutic targeting of one pathway does not inadvertently exacerbate harmful inflammation or suppress beneficial responses. Future studies integrating selective pathway modulation with precise temporal control, and employing relevant disease models, will be critical for defining these interactions.

Ultimately, determining whether succinate alone or in combination with cytokines and chemokines can be harnessed to amplify protective immunity or dampen pathogenic inflammation remains an important and exciting avenue for future research.
